# Muscle reflex in heart failure: the role of exercise training

**DOI:** 10.3389/fphys.2012.00398

**Published:** 2012-10-05

**Authors:** Han-Jun Wang, Irving H. Zucker, Wei Wang

**Affiliations:** Department of Cellular and Integrative Physiology, University of Nebraska Medical CenterOmaha, NE, USA

**Keywords:** physical training, myocardial infarction, muscle afferents, exercise, sympathetic nerve activity

## Abstract

Exercise evokes sympathetic activation and increases blood pressure and heart rate (HR). Two neural mechanisms that cause the exercise-induced increase in sympathetic discharge are central command and the exercise pressor reflex (EPR). The former suggests that a volitional signal emanating from central motor areas leads to increased sympathetic activation during exercise. The latter is a reflex originating in skeletal muscle which contributes significantly to the regulation of the cardiovascular and respiratory systems during exercise. The afferent arm of this reflex is composed of metabolically sensitive (predominantly group IV, C-fibers) and mechanically sensitive (predominately group III, A-delta fibers) afferent fibers. Activation of these receptors and their associated afferent fibers reflexively adjusts sympathetic and parasympathetic nerve activity during exercise. In heart failure, the sympathetic activation during exercise is exaggerated, which potentially increases cardiovascular risk and contributes to exercise intolerance during physical activity in chronic heart failure (CHF) patients. A therapeutic strategy for preventing or slowing the progression of the exaggerated EPR may be of benefit in CHF patients. Long-term exercise training (ExT), as a non-pharmacological treatment for CHF increases exercise capacity, reduces sympatho-excitation and improves cardiovascular function in CHF animals and patients. In this review, we will discuss the effects of ExT and the mechanisms that contribute to the exaggerated EPR in the CHF state.

## Introduction

A hallmark of patients suffering from chronic heart failure (CHF) is exercise intolerance characterized by fatigue and shortness of breath during exercise (Francis, 1985; Cohn, 1990; Sullivan et al., 1990; Wilson, 1995). The degree of exercise intolerance is important to characterize in patients with CHF, since it has implications for morbidity, disability, and prognosis, and is often the reason a patient seeks medical attention. Originally, the explanation for exercise intolerance in CHF appears to be mainly due to inadequate delivery of oxygen from the failing heart to the working muscle. However, evidence suggests that the degree of exercise intolerance is not directly related to the degree of cardiac dysfunction (Franciosa et al., 1981; Sullivan and Hawthorne, 1995). Rather, it is generally thought that the factors in the peripheral musculature may play a critical role in mediating exercise intolerance. These peripheral factors include abnormalities in endothelial function, vasodilatory capacity, changes in skeletal muscle structure, oxidative enzyme activity and a reflex originating in skeletal muscle (termed “exercise pressor reflex,” EPR) that contributes significantly to the regulation of the cardiovascular and respiratory function during exercise (Myers and Froelicher, 1991; Sullivan and Hawthorne, 1995; Myers et al., 1999; Piepoli et al., 1999). In CHF patients, this reflex is exaggerated and causes extreme activation of the sympathetic nervous system even during moderate exercise. Exaggerated sympathetic activation by the EPR during exercise likely restrains muscle blood flow, arteriolar dilatation, and capillary recruitment, leading to under perfused areas of working muscle. In addition to vasoconstriction in skeletal muscle, hyperventilation is another consequence of the exaggerated EPR during exercise, both of which accentuates the symptoms of exercise intolerance. It is important to understand how the exaggerated EPR contributes to the exercise intolerance in CHF patients. Furthermore, the exaggerated sympatho-excitation that occurs during exercise also increases the risk of experiencing myocardial ischemia, myocardial infarction, cardiac arrest, and/or stroke during or immediately after exercise in these patients.

As exercise intolerance and exaggerated sympatho-excitation are important clinical features in these patients, therapeutic interventions are largely aimed at improving these symptoms. A particular interest has recently been directed toward the exaggerated EPR in CHF (Piepoli et al., 1996, 1999; Khan and Sinoway, 2000; Piepoli, 2006; Wang et al., 2010b, 2012). Once thought to be contraindicated in patients with CHF, long-term regular exercise training (ExT for at least 8 weeks) as a non-pharmacological treatment for CHF is now commonly employed in these patients, and has been shown to increase the quality of life as well as survival (Belardinelli et al., 1999; Piepoli et al., 2004; Smart and Marwick, 2004; Jankowska et al., 2007; Wisloff et al., 2007; Flynn et al., 2009; O'Connor et al., 2009). The beneficial effects of ExT include improved autonomic balance, reduced neurohumoral activation, increase in exercise capacity and ameliorated myopathy in CHF patients and animals (Pliquett et al., 2003; Roveda et al., 2003; Rondon et al., 2006; Jankowska et al., 2007; Mueller, 2007b; Negrao and Middlekauff, 2008). Adequate discussion of the beneficial effects of ExT in CHF is a large endeavor and beyond the scope of the current review. Therefore, this review will be narrowed and focus on the role of ExT in improving the exaggerated EPR in CHF. Furthermore, we will also discuss the mechanisms underlying the beneficial effect of ExT on the exaggerated EPR in CHF.

## Sympathetic activation during exercise

During exercise the sympathetic nervous system is activated, which results in an increase in arterial pressure (AP), heart rate (HR), and peripheral vasoconstriction, especially to non-exercising tissues. Two theories have been postulated to explain the increases in cardiovascular and ventilatory function during exercise: central command and the EPR. Central command is a mechanism whereby neural motor and sympathetic activation occur in parallel, i.e., a volitional signal from the motor cortex or subcortical nuclei, responsible for recruiting motor units, activate cardiovascular control areas in the brainstem to modulate sympathetic and parasympathetic activity during exercise (Goodwin et al., 1972; Eldridge et al., 1985). It has been suggested that this system is linked to skeletal muscle metabolic needs via parallel rostral brain activation of motor and autonomic centers. These autonomic adjustments elicit changes in ventilation, HR and AP proportional to the intensity of exercise. The EPR is a peripheral neural reflex originating in skeletal muscle which contributes to the regulation of the cardiovascular and respiratory systems during physical activity. This reflex is essential for the maintenance of adequate blood perfusion to the exercising muscle, thereby matching the metabolic demands that exercise creates (McCloskey and Mitchell, 1972).

## The exercise pressor reflex

Although several reviews have been published describing the EPR (Sinoway and Li, 2005; Smith et al., 2006a; Murphy et al., 2011), a brief synopsis is warranted here. Alam and Smirk (1937) were the first to offer evidence suggesting that chemical byproducts of muscle contraction could evoke a pressor reflex. These authors demonstrated that dynamic calf exercise evoked increases in BP and HR that were maintained by circulatory arrest at the end of exercise. These findings provided the earliest evidence that the accumulation of metabolites in the contracting muscle elicited the EPR. A study by McCloskey and Mitchell (1972) demonstrated that anodal blockade of the L7–S1 dorsal roots of the cat blocked thickly myelinated group I and II afferents but did not affect the cardiovascular responses to contraction whereas topical application of a local anesthetic to the dorsal roots did not block group I and II afferents but did abolish the cardiovascular responses to contraction, indicating that activation of this reflex is mediated by stimulation of thinly myelinated group III and IV but not thickly myelinated group I and II afferents. Studies by Kaufman et al. (1983, 1984) demonstrated that group III fibers in the triceps surae muscle of the cat are predominantly mechanically sensitive, whereas unmyelinated group IV muscle afferents are chemically sensitive.

Anatomically, group III nerve endings terminate in the collagenous connective tissue, the endoneurium of the triceps surae and calcaneal tendon of the cat, which are rapidly excited by mechanical deformation of their receptive field and then quickly adapted during the steady state period of muscle contraction (Kniffki et al., 1978; Kaufman et al., 1983, 1984; Andres et al., 1985; Mense and Meyer, 1985; Hayward et al., 1991; Adreani et al., 1997; Adreani and Kaufman, 1998). As such, receptors associated with these afferent fibers are termed “mechanoreceptors,” although a few are responsive to chemical stimuli. On the other hand, sensory receptors associated with group IV afferent neurons are located on unencapsulated nerve endings that terminate within the walls of capillaries, venules, and lymphatic vessels of skeletal muscle, which are predominately excited by the accumulation of metabolites produced by contracting muscle (Kaufman et al., 1983; Andres et al., 1985). With regard to the time needed for accumulation of metabolites, activation of group IV afferents are always delayed (5–20 s) following muscle contraction (Kaufman et al., 1983; Mense and Meyer, 1985). Sensory receptors associated with group IV afferents are termed “metaboreceptors” although a few are also responsive to mechanical stimuli.

**Figure 1:**
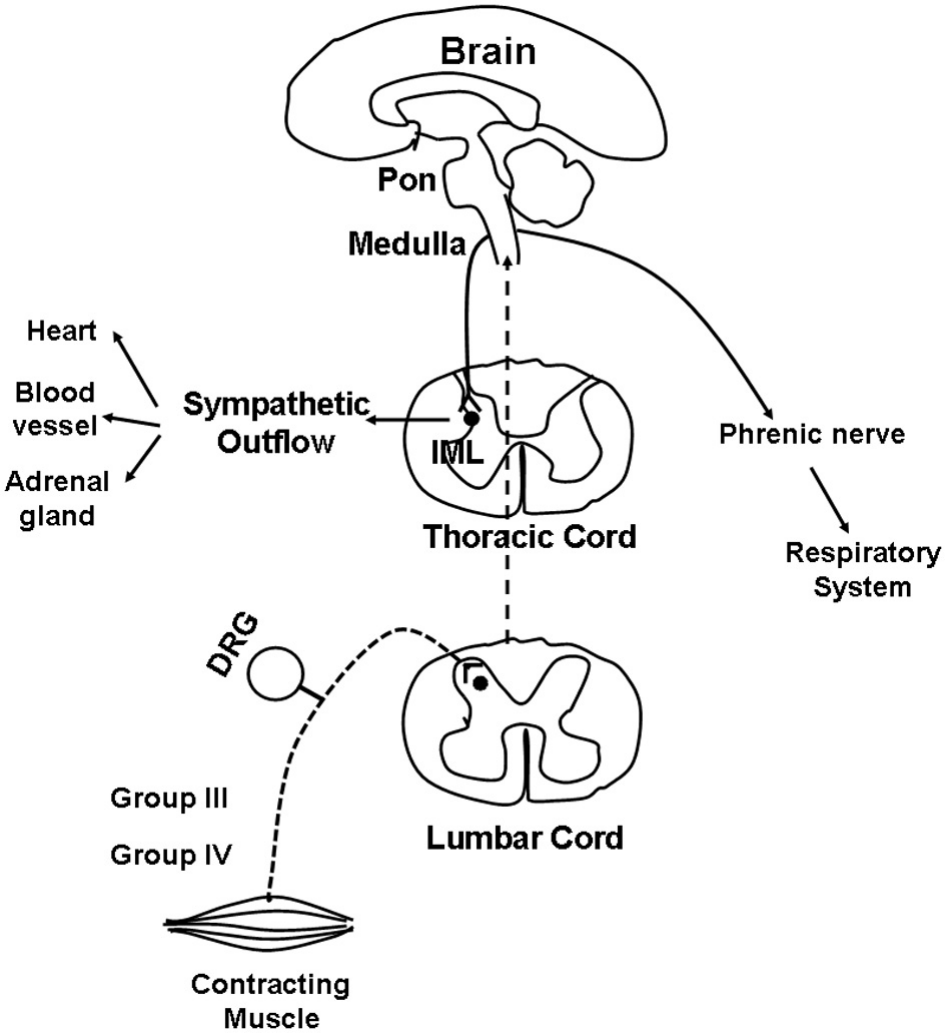
**Schematic illustrating the proposed pathway for the reflex cardiovascular and respiratory changes evoked by skeletal muscle contraction.** Dashed lines indicate the afferent limb of this reflex pathway whereas solid lines points out the efferent limb. See text for additional discussion.

The first site of synapse for most muscle group III and IV afferents is the dorsal horn of the spinal cord, specifically Rexed's laminae I, II, V, and X (Kalia et al., 1981; Mense and Craig, 1988; Li and Mitchell, 2002; Wilson et al., 2002). Although the specific pathway remains unknown, muscle afferents project from the dorsal horn to the brain stem along a path that includes the dorsolateral sulcus and the ventral spinal cord (Iwamoto et al., 1984; Kozelka and Wurster, 1985; Dykes and Craig, 1998). From the dorsal horn, ascending projections activate cells in the medulla. Above the medulla, the central integration of the pressor reflex is complex, involving multiple regions. However, evidence suggests that full expression of the EPR at least requires an intact ponto-medullary region of the brainstem (Iwamoto et al., 1985). Those nuclei responsive to EPR stimulation have been described in the nucleus tractus solitarius (NTS), rostral ventral medulla, caudal ventrolateral medulla, lateral tegmental field, nucleus ambiguus, and the ventromedial region of the rostral periaqueductal grey (Iwamoto et al., 1982; Iwamoto and Kaufman, 1987; Li et al., 1997; Li and Mitchell, 2000). From the medulla, descending projections synapse on sympathetic pre-ganglionic neurons in the intermediolateral cell columns (IML) of the spinal cord and then project to the synapse at the paravertebral sympathetic chain ganglia, and finally innervate the heart and vasculature. The EPR-mediated adjustments in parasympathetic and SNA result in increases in cardiac contractility, SV, HR, and BP (Longhurst and Mitchell, 1979; Murata and Matsukawa, 2001; Koba et al., 2006; Wang et al., 2010b). It is through the pathways, outlined in Figure 1, that skeletal muscle reflexes contribute to cardiovascular and respiratory regulation during physical activity.

## Abnormalities of exercise pressor reflex in CHF

There is general agreement that the EPR is exaggerated in humans with CHF and that these exaggerations correlate with morbidity and mortality as well as with decreased left ventricular (LV) function (McClain et al., 1993; Middlekauff et al., 2000, 2001; Piepoli and Coats, 2007; Piepoli et al., 2008). The emerging evidence describing dysfunction of the reflex with the advent of CHF has been highlighted in several recent reviews (Sinoway and Li, 2005; Smith et al., 2006a; Garry, 2011; Murphy et al., 2011). Despite this, defining the mechanisms that mediate the abnormal EPR in CHF patients has proven to be difficult. For example, studies in human subjects have not been able to clearly discern whether peripheral primary afferent neurons or central areas that process the EPR are responsible for the exaggerated EPR that is observed in CHF. In addition, the literature surrounding the issue defining the contribution of metabo- or mechano-reflex to the exaggerated EPR in CHF patients is conflicted. A great deal of controversy exists regarding the contribution of the metabolic component of the EPR (metaboreflex) because its activity has been reported to be both enhanced (Piepoli et al., 1996, 2008; Piepoli and Coats, 2007) and reduced (Sterns et al., 1991; Middlekauff et al., 2000) in response to exercise in CHF patients. Based on measurements of ventilation, the studies of Piepoli et al. (Piepoli et al., 1996, 2008; Piepoli and Coats, 2007) showed that CHF patients had an overactive metaboreflex compared with control subjects. However, based on measurements of blood pressure or sympatho-excitatory responses to post-contraction circulatory arrest (PCCA, an isolated activator of the muscle metaboreflex), the studies of Sterns et al. (1991) and Middlekauff et al. (2000) showed that metaboreflex function is blunted rather than exaggerated in this disease state. The discrepant conclusions that the metaboreflex is blunted or exaggerated in CHF appear to be due to different measurements of physiologic parameters such as ventilation, blood pressure and sympathetic nerve activity. In addition, a central command mechanism, which cannot absolutely be excluded in human studies, may also contribute to this discrepant conclusion. Compared to studies concerning the metaboreflex in CHF patients, the studies focusing on the role of the mechanoreflex in mediating the exaggerated EPR is generally consistent, suggesting that an overactive mechanoreflex contributes to the exaggerated EPR in CHF patients. McClain et al. (1993) reported that limb congestion, a common feature of congestive CHF, increases the sympathetic nerve response to handgrip exercise. Moreover, subsequent studies from the same laboratory demonstrated that limb congestion could sensitize muscle mechanoreceptors and in the process increase synchrony between contraction and sympathetic discharge (Mostoufi-Moab et al., 2000). Middlekauff et al. (2001) suggesting that reflex renal vasoconstriction is exaggerated in both magnitude and duration during dynamic exercise in HF patients. Moreover, subsequent studies from this laboratory have shown that the mechanoreflex is exaggerated in humans suffering from CHF which is most likely due to sensitization of the mechanoreceptor afferents by cyclooxygenase products (Middlekauff and Chiu, 2004; Middlekauff et al., 2004).

**Figure 2:**
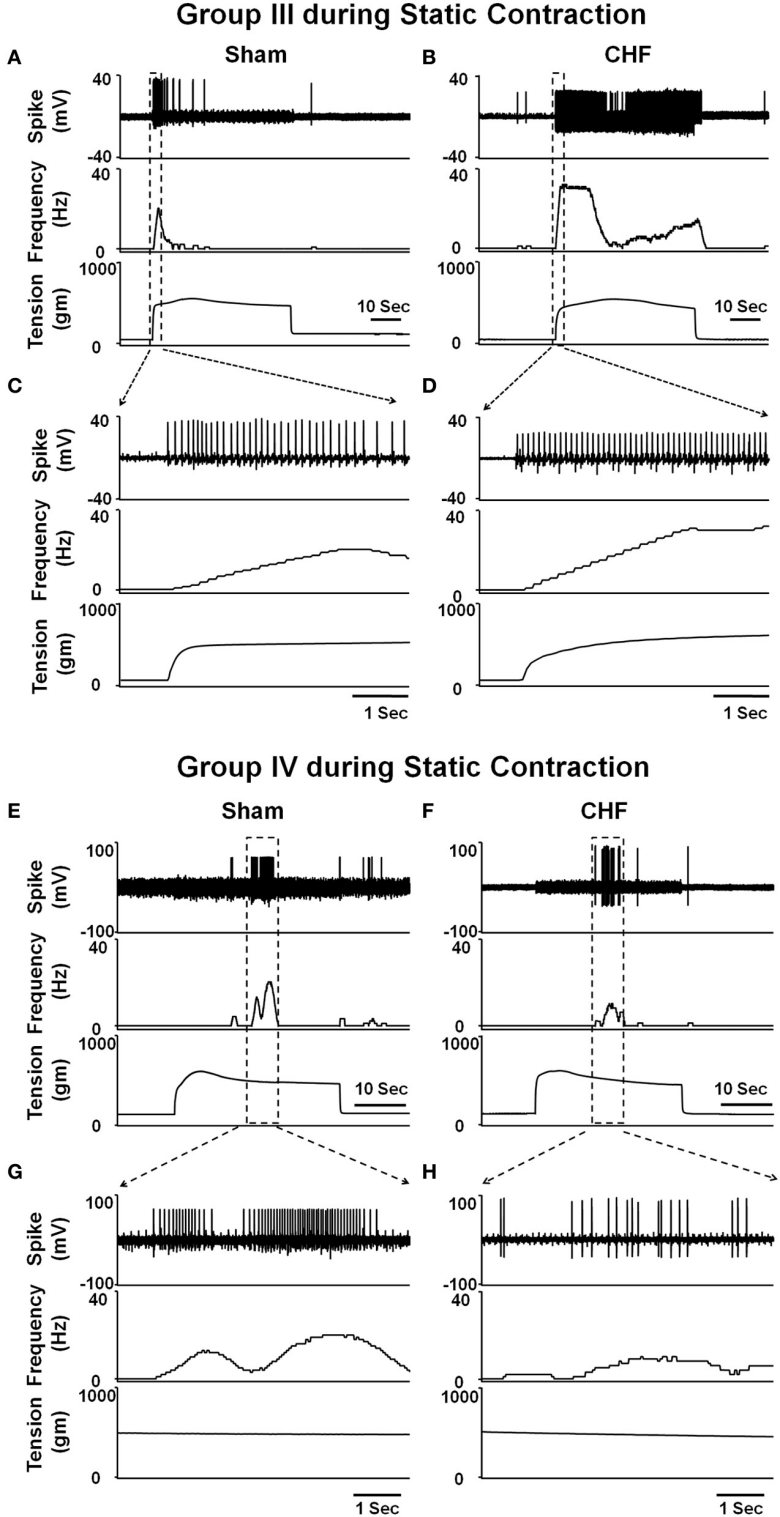
**Representative recordings showing the discharge of group III (A–D) and IV (E–H) afferents in response to static contraction induced by electrical stimulation of L5 ventral root in sham (Group III in panel (A): CV, 5.2 m/s; Group IV in panel (E): CV, 0.8 m/s) and CHF rats (Group III in panel (B): CV, 7.0 m/s; Group IV in panel (F): CV, 0.5 m/s). (C)** and **(D)**, 5-s recording of two group III fibers discharge derived from the broken-lined box in **(A)** and **(B)**, respectively. **(G)** and **(H)**, 6-s recording of two group IV fibers discharge derived from the broken-lined box in **(E)** and **(F)**, respectively. [Reprinted from Wang et al. (2010a). Copyright @ 2010 The Physiological Society. Used with permission.]

**Figure 3:**
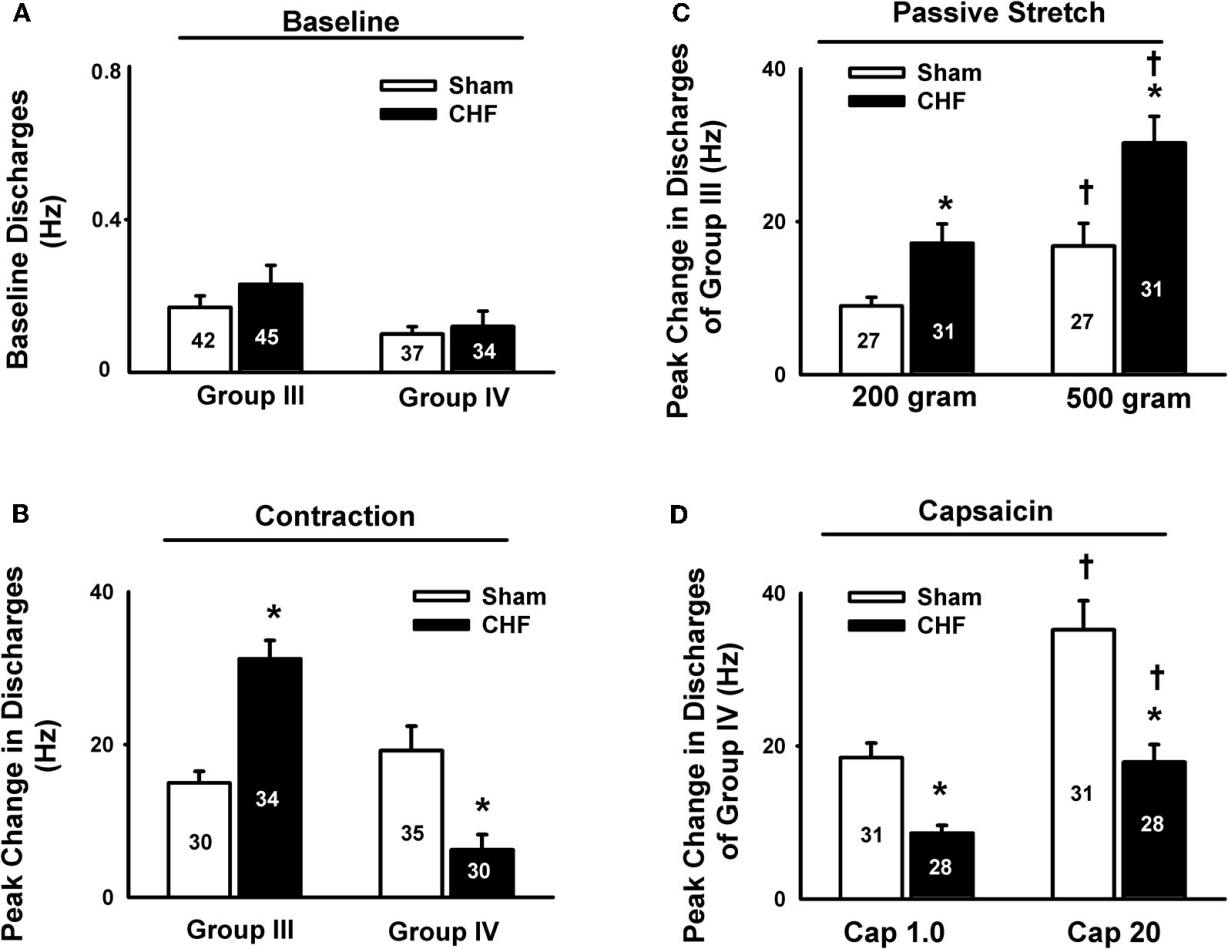
**Mean data showing the baseline discharge of group III and IV afferents in sham and CHF rats (A) and the responses of group III and IV afferents to static contraction induced by electrical stimulation of L5 ventral root in sham and CHF rats (B).** Mean data showing the discharge of group III and IV afferents in response to two levels of passive stretch **(C)** and two doses of capsaicin **(D)** respectively in sham and CHF rats. Data are expressed as Mean ± SE. ^*^P < 0.05 vs. sham, ^†^*P* < 0.05 vs. lower level of stretch or lower dose of capsaicin. [Reprinted from Wang et al. (2010a). Copyright @ 2010 The Physiological Society. Used with permission.]

In animal studies, using a decerebrate rat model, Smith et al. (2003, 2005a,b) conducted a series of convincing experiments designed specifically to examine EPR function in CHF and to determine the contribution of the muscle mechanoreflex and metaboreflex to the EPR in this disease. Their findings suggest (1) that the overactive cardiovascular response to exercise in CHF is mediated, in part, by an exaggerated EPR; (2) that the muscle metaboreflex is blunted and that the muscle mechanoreflex is enhanced in CHF; (3) that the mechanoreflex mediates the exaggerated EPR activity observed in CHF; (4) that the decreased sensitivity of group IV afferent neurons is important to the development of EPR hyperactivity. In parallel studies, Li et al. (2004) also reported a similar finding as that of Smith et al. (2003, 2005a,b), showing that the muscle metaboreflex control of cardiovascular activity is blunted and that the muscle mechanoreflex is enhanced in rats with large myocardial infarcts. Subsequent studies from the same laboratory (Koba et al., 2008) showed that renal and lumbar sympathetic nerve responses to muscle contraction were larger in CHF rats than in healthy control rats, indicating that the EPR contributes to the exaggerated sympatho-excitation during exercise. Recently, using the technique of single fiber recording, we (Wang et al., 2010a) demonstrated that the responses of group III afferents to contraction and stretch were enhanced in rats with dilated cardiomyopathy (induced by ligation of the left anterior descending coronary artery) whereas the responses of group IV afferents to contraction and capsaicin were reduced in these rats compared to sham-operated rats (Figures 2 and 3), which provide direct evidence that the exaggerated EPR in CHF is, at least in part, due to the peripheral sensitization of muscle mechanically sensitive afferents. However, the EPR is a multisynaptic reflex involving the following: (1) the receptors activating the afferent fibers; (2) primary afferent neurons, (3) second-order spinal neurons, (4) neurons in medullary centers, (5) sympathetic and parasympathetic efferent neurons, and (6) the end organs that the efferent fibers innervate. Whether other parts of this reflex arc are also involved in the genesis of the exaggerated EPR in the CHF state remains unclear. Further studies are worthy of being carried out to address this issue.

## Effect of ExT on the EPR in health and CHF

Over the past decade numerous clinical trials and small randomized studies have demonstrated that long-term regular exercise is safe in stable CHF patients and increases the quality of life as well as survival (Belardinelli et al., 1999; Khan and Sinoway, 2000; Jankowska et al., 2007; Mueller, 2007a; Wisloff et al., 2007). Therefore, ExT has been recommended as a non-pharmacological treatment for CHF, ischemic heart disease and hypertension by the American Heart Association (Fletcher et al., 1996; Halbert et al., 1997; Fletcher et al., 2001; Pina et al., 2003). Furthermore, several clinical and experimental studies have also suggested that ExT effects EPR function in health and disease states. However, the mechanisms underlying the effect of ExT on EPR function in both health and disease remain largely unknown.

### Effect of ExT on the EPR in health

**Figure 4:**
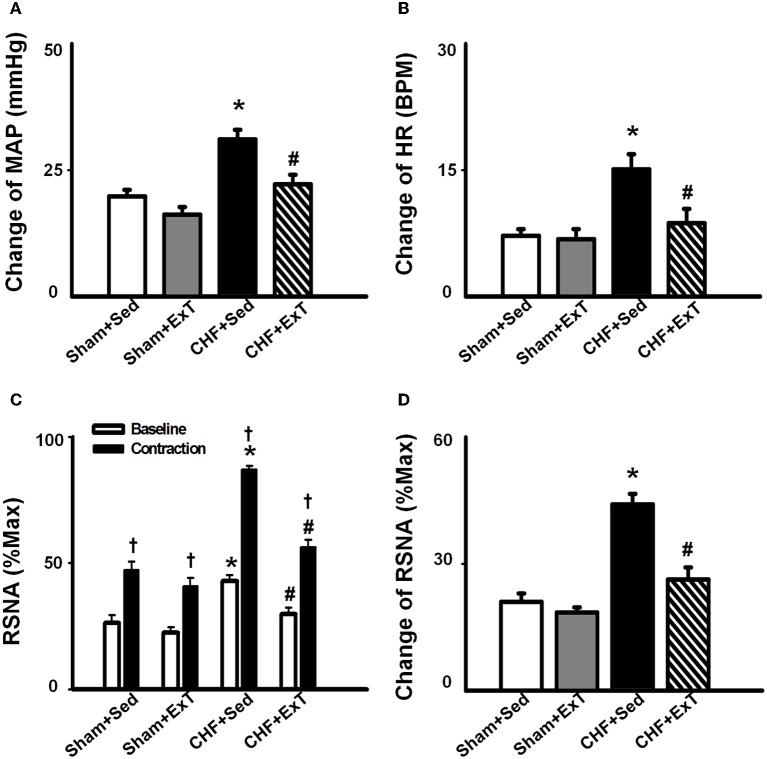
**Mean data showing the effects of ExT on pressor (A), heart rate (B) and sympatho-excitatory (D) responses to a 30-s static contraction in sham and CHF rats.** Both baseline and contraction-induced RSNA were compared in sham+Sed, sham+ExT, CHF+Sed and CHF+ExT rats (C). Values are Mean ± SE, ^*^P < 0.05 vs. sham+Sed and sham+ExT, ^†^*P* < 0.05 vs. baseline RSNA, #*P* < 0.05 vs. CHF+Sed. [Reprinted from Wang et al. (2010b). Copyright @ 2010 American Heart Association. Used with permission.]

**Figure 5:**
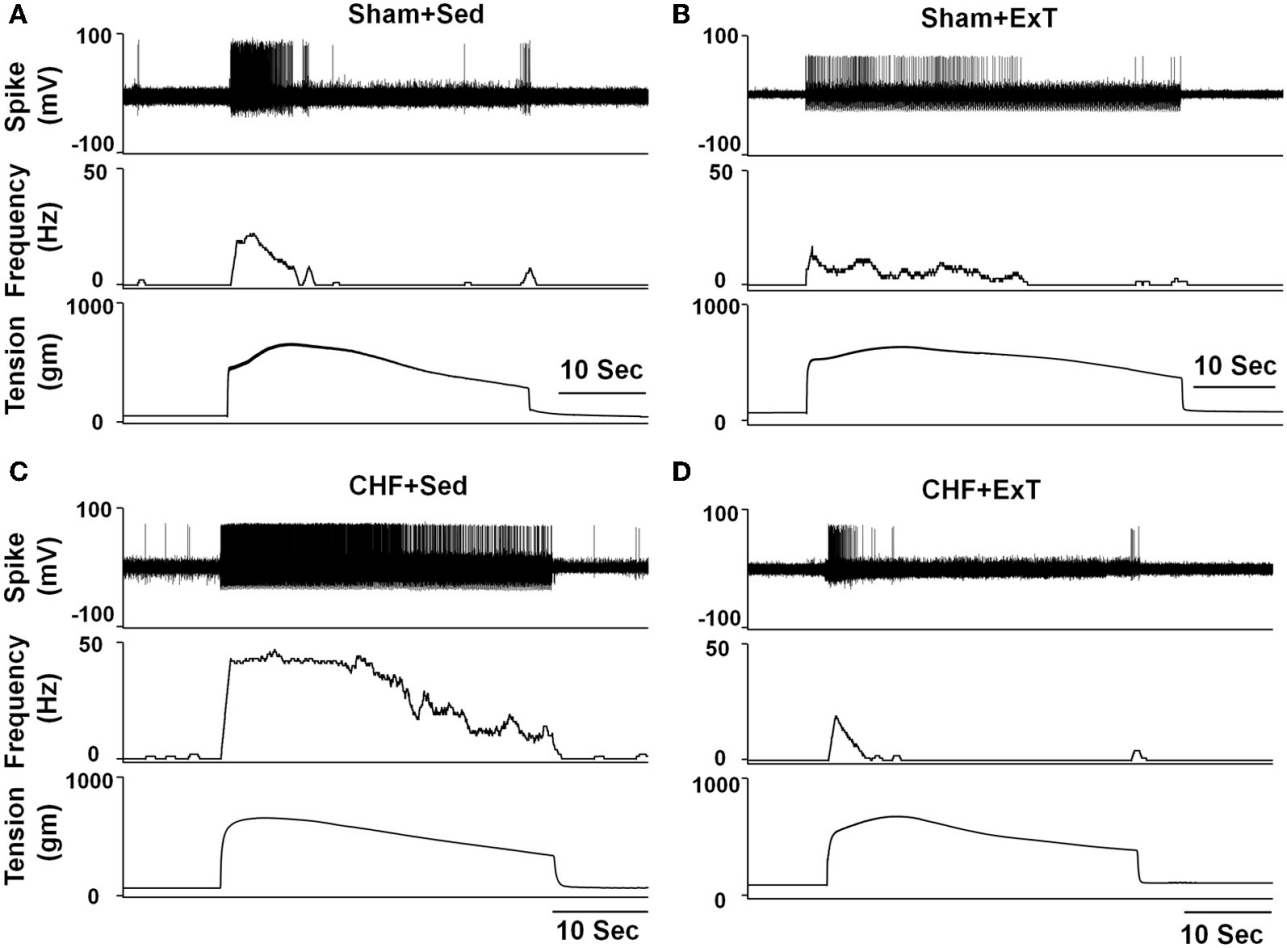
**Representative recordings showing the discharge of group III afferents in response to static contraction induced by electrical stimulation of L5 ventral root in sham+Sed (CV, 4.1 m/s, A), Sham+ExT(CV, 3.0 m/s, B), CHF+Sed (CV, 4.7 m/s, C) and CHF+ExT rats (CV, 5.3 m/s, D).** [Reprinted from Wang et al. (2012). Copyright @ 2012 American Heart Association. Used with permission.]

**Figure 6:**
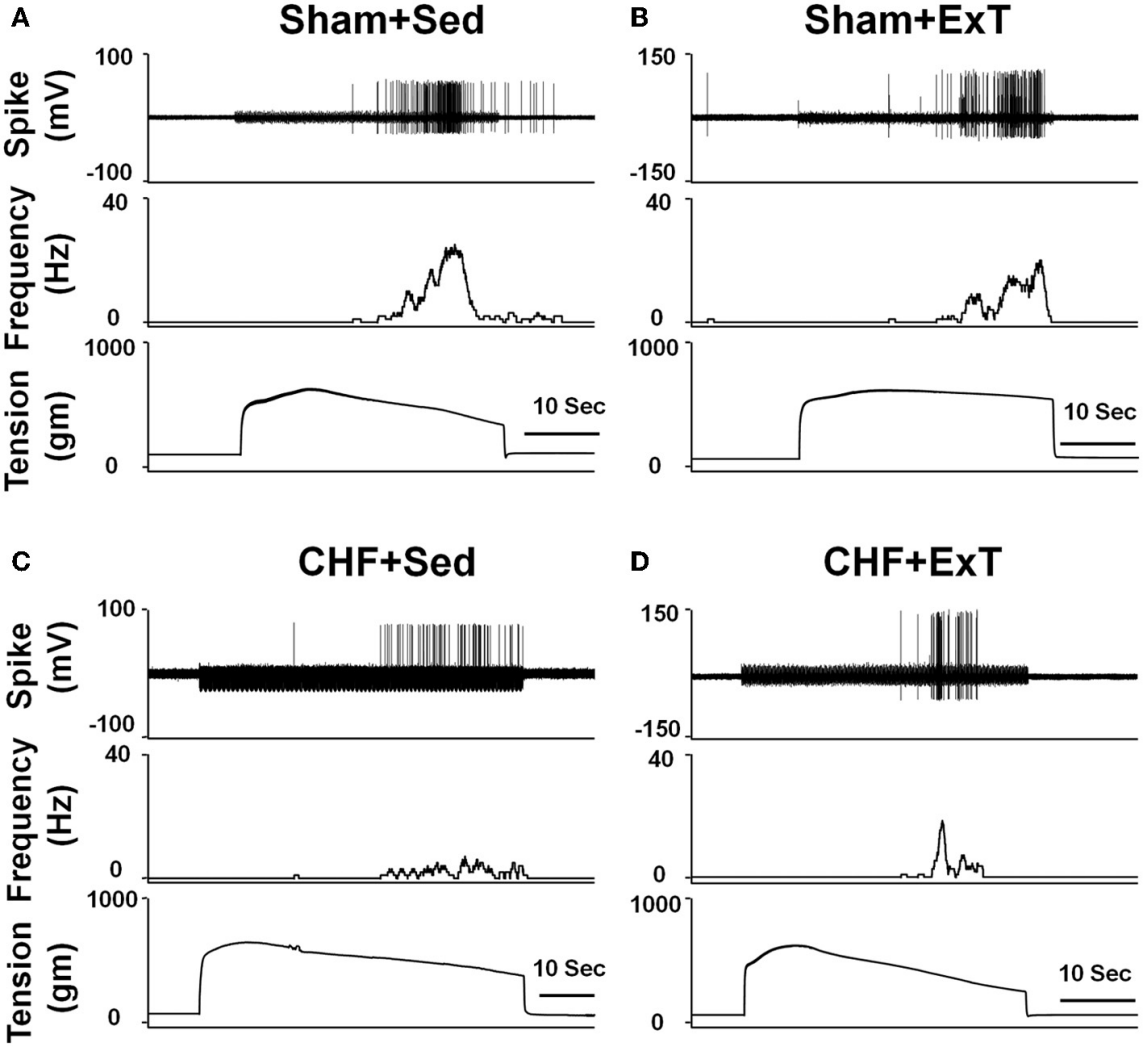
**Representative recordings showing the discharge of group IV afferents in response to static contraction induced by electrical stimulation of L5 ventral root in sham+Sed (CV, 1.2 m/s, A), Sham+ExT(CV, 0.82 m/s, B), CHF+Sed (CV, 0.73 m/s, C) and CHF+ExT rats (CV, 1.0 m/s, D).** [Reprinted from Wang et al. (2012). Copyright @ 2012 American Heart Association. Used with permission.]

**Figure 7:**
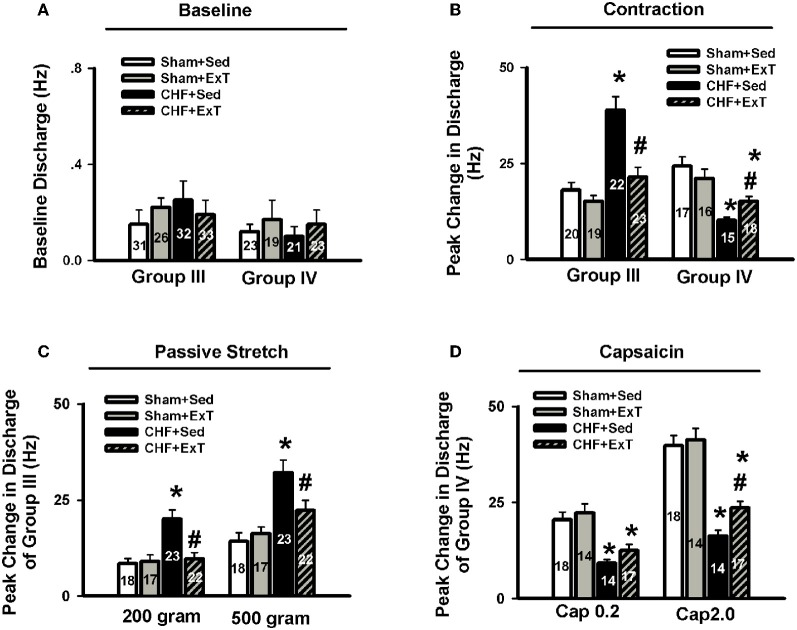
**Mean data showing the baseline discharge of group III and IV afferents (A) and the responses of group III and IV afferents to static contraction induced by electrical stimulation of L5 ventral root (B) in Sham+Sed, Sham+ExT, CHF+Sed and CHF+ExT rats.** Mean data showing the discharge of group III and IV afferents in response to two levels of passive stretch **(C)** and two doses of capsaicin **(D)** respectively in Sham+Sed, Sham+ExT, CHF+Sed and CHF+ExT rats. The digit in the bar graph indicates the number of recording fibers. Data are expressed as Mean ± SE. ^*^P < 0.05 vs. sham + Sed, #*P* < 0.05 vs. CHF + Sed. [Reprinted from Wang et al. (2012). Copyright @ 2012 American Heart Association. Used with permission.]

An earlier study by Sinoway et al. (1996) reported that 4-week forearm training reduced sympathetic responses and mean AP rises during rhythmic voluntary handgrip exercise in normal subjects. Following study from this group (Mostoufi-Moab et al., 1998) demonstrated that forearm exercise conditioning paradigm also attenuated the pressor response to ischemic rhythmic exercise and decreased lactate accumulation and venous pH values, suggesting that muscle metaboreceptor afferent activity may be reduced because of a decrease in metabolite accumulation in the trained muscle. However, during such voluntary exercise, it has been difficult to distinguish between possible training-induced changes in central command, and the different muscle afferent inputs to the response. Therefore, Fisher and White (1999) used two exercise modes to re-evaluate the effects of ExT on central command and the EPR in healthy subjects. The first exercise mode was voluntary muscle contraction, which potentially involves central command as well as muscle mechanoreceptor and muscle metaboreceptor stimulation, and the second was electrically evoked contraction (involuntary) at the same force level. In this instance, central command was removed but muscle receptor activity should remain at the same level as in the voluntary exercise mode. Both forms of exercise were followed by PCCA where muscle metaboreceptor activity predominates. These data demonstrated that 6-week calf muscle training had no effect on the muscle afferent input to the pressor response to electrically evoked contraction in the untrained limb, since cardiovascular responses were unchanged both during exercise and PCCA. However, during voluntary contraction of the untrained limb, diastolic blood pressure and HR rises were attenuated after training, but neither were altered from pre-training values during PCCA. Therefore the changes can only be explained by decreased central command during exercise. In animal experiments, we (Wang et al., 2010b) recently reported that although 8–10 week of treadmill ExT tended to reduce the blood pressure, HR and renal sympathetic activation responses to involuntary static contraction by electrical stimulation of ventral roots in a decerebrate rat model where central command was removed, this training effect did not reach statistical significance (Figure 4), indicating that ExT has less effect on the EPR in healthy animals. Direct evidence from muscle afferent recording experiments (Wang et al., 2012) also supports that training has less effect on the sensitivity of group III and IV afferents in healthy rats (Figures 5–7). Taken together the evidence suggests that it is very likely that ExT attenuates cardiovascular activity during exercise mainly via affecting central command rather than muscle afferent input in normal subjects or animals.

### Effect of ExT on the EPR in CHF

**Figure 8:**
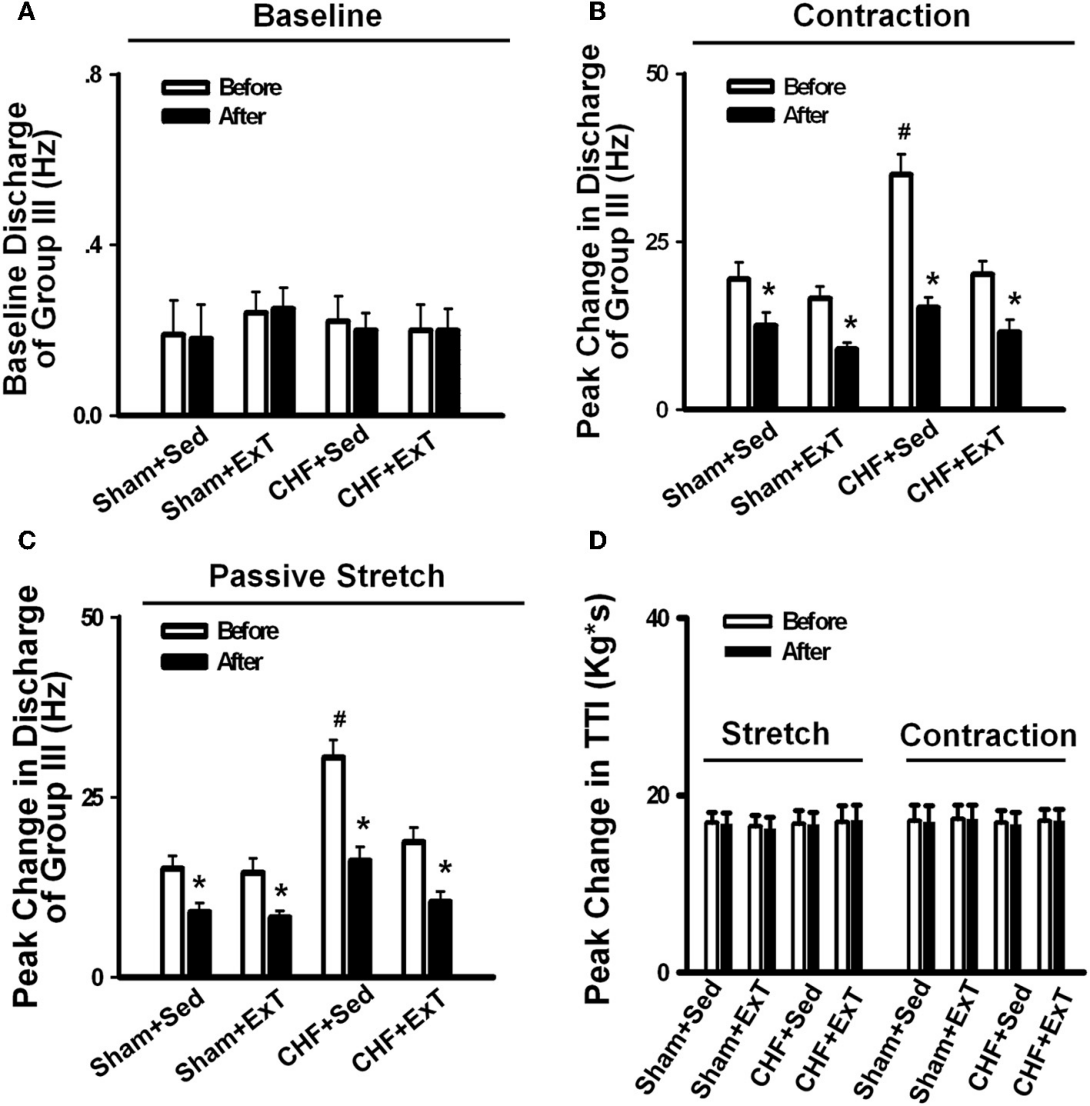
**Mean data showing the effect of PPADS, a P2X antagonist, on the baseline discharge (A) and the responses of group III afferents to either static contraction (B) or passive stretch (C) in Sham+Sed, Sham+ExT, CHF+Sed, and CHF+ExT rats. (D)** The effect of PPADS on the TTI produced by passive stretch or static contraction in Sham+Sed, Sham+ExT, CHF+Sed and CHF+ExT rats. Data are expressed as Mean ± SE. *n* = 8 − 10/each group. ^*^P < 0.05 vs. before, #*P* < 0.05 vs. Sham+Sed. [Reprinted from Wang et al. (2012). Copyright @ 2012 American Heart Association. Used with permission.]

The beneficial effect of ExT on the exaggerated EPR in CHF has also been previously demonstrated. Piepoli et al. (1996) reported that in patients with CHF (8–38 months, New York Heart Association (NYHA) class II–III), there is an exaggerated exercise-evoked sympatho-excitation, vasoconstrictor, and ventilatory drive characteristic of this population of patients, which is partially reversed by 6-week forearm training. These findings indicate a potential beneficial effect of ExT on the abnormal muscle reflex function in CHF patients. However, due to intrinsic limitations of human research, the study of Piepoli et al. could not distinguish between possible training-induced changes in central command, and the different muscle afferent inputs to the response. In animal experiments, using a decerebrate model to remove the cortical structures from which central command originates, we recently (Wang et al., 2010b) observed that 8–10 weeks of treadmill ExT initiated at an early stage of CHF (i.e., 2 weeks after coronary ligation) prevents the exaggerated HR, pressor and sympatho-excitatory responses to either static contraction or passive stretch (a purely mechanical stimulus) and partially prevents the blunted cardiovascular responses to injection of exogenous capsaicin (a chemical stimulus) in CHF rats (Figure 4). These findings indicate that ExT at an early stage of CHF has a beneficial effect on the exaggerated EPR. More recently, we (Wang et al., 2012) further demonstrated that this training protocol prevents the sensitization of group III afferents and partially prevents the blunted sensitivity of group IV afferents in CHF rats (Figure 8), suggesting that the beneficial effects of ExT on the exaggerated EPR is at least, in part, mediated by preventing the abnormal sensitization of muscle afferents in the CHF state.

## Mechanisms underlying the beneficial effect of ExT on the exaggerated EPR in CHF

Although the beneficial effects of ExT on the exaggerated EPR has been demonstrated in CHF patients and animals (Piepoli et al., 1996; Piepoli, 2006; Wang et al., 2010b, 2012), the underlying mechanisms have not been completely identified. For example, in addition to muscle afferents, whether other components of the reflex arc are also affected by ExT in CHF remains unknown. We previously demonstrated that ExT also attenuated the exaggerated sympatho-excitation at rest in CHF animals (Liu et al., 2000; Gao et al., 2007). The effects of ExT on sympatho-excitation in CHF is, in part, mediated by effects on central neural structures such as the rostral ventrolateral medulla (RVLM) and the nucleus tractus solitaries (NTS) (Mueller and Hasser, 2006; Gao et al., 2007). Therefore, it is possible that ExT affects the EPR in CHF via a central mechanism. In addition, in a previous study (Gao et al., 2007) we demonstrated that the effects of ExT on central neural structures such as the RVLM also contribute to an improvement of the blunted arterial baroreflex function in CHF. The latter and the EPR are well known to modify one another functionally during exercise. For example, it has been demonstrated that the cardiovascular response to activation of the EPR is enhanced in normotensive baro-denervated cats and rats (Waldrop and Mitchell, 1985; Smith et al., 2006b). Therefore, we speculate that a blunted baroreflex in CHF might contribute to the genesis of the exaggerated EPR whereas the ExT-mediated improvement of the blunted arterial baroreflex might ameliorate the exaggerated EPR. However, direct evidence for these hypotheses is absent.

Previous studies (Drexler et al., 1992; Mancini et al., 1992) have shown that peripheral skeletal myopathy develops in CHF (e.g., muscle atrophy, decreased peripheral blood flow, fiber-type transformation and reduced oxidative capacity). These abnormalities in the peripheral musculature in CHF may alter the environment around muscle afferent endings to sensitize muscle afferents. Because ExT has been reported to reverse skeletal myopathy in CHF (Howald et al., 1985; Hambrecht et al., 1997), this effect may play a critical role in the ExT-mediated improvement of the abnormal sensitization of muscle afferents in CHF. In addition, a chronic reduction in skeletal muscle perfusion in CHF patients may alter muscle metabolism and cause excessive accumulation of metabolites during exercise. As such, the potential chronic exposure to excess metabolites could result in the sensitization of muscle afferents. It has been well documented that ExT leads to an increased perfusion of skeletal muscle in CHF patients (De Matos et al., 2004). This combined with an increased ability of the muscle to maintain aerobic metabolism leads to a decreased reliance on anaerobic metabolism. We speculate that this will lead to lower interstitial concentration of metabolites, evoking less muscle afferent stimulation.

### ExT reversed muscle type shift in CHF

Iwamoto and Botterman (1985) reported that contraction of fast-twitch fibers (type II) evoked a larger pressor response to static contraction compared to slow-twitch fiber (type I) contraction. Furthermore, the study by Wilson et al. (1995) demonstrated that chronic low-frequency electrical stimulation of the tibial nerve of one hindlimb of adult rabbits, which converted the gastrocnemius (predominately type II) to a muscle that was primarily type I, decreased the pressor response to static contraction. These two studies suggested that type II fiber contraction may activate a larger number of muscle afferent receptors. In the CHF state, a muscle fiber-type shift from type I to type II could cause an exaggerated EPR. Since muscle fiber-type transformation in CHF can be reversed by ExT (Howald et al., 1985; Hambrecht et al., 1997), the improvement of abnormal fiber-type shift by ExT may subsequently affect muscle afferent function, and eventually ameliorate the exaggerated EPR function in the CHF state. Clearly, further research is needed in this area.

### Purinergic receptors are involved in the mechanism by which ExT prevents the sensitization of group III afferents in CHF

**Figure 9:**
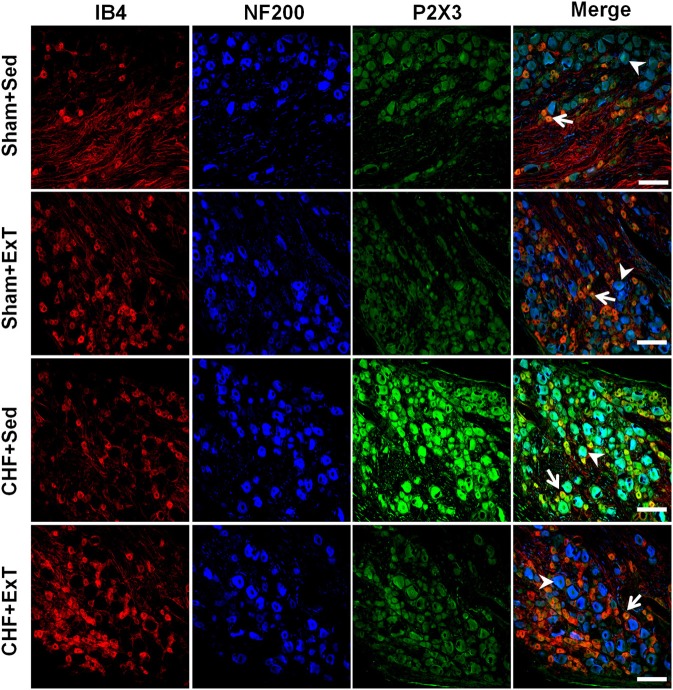
**Immunohistochemical data showing the protein expression of P2X3 receptors in L4/L5 dorsal root ganglion (DRG) in Sham+Sed, Sham+ExT, CHF+Sed and CHF+ExT rats.** Isolectin B4 (IB4), a C-fiber neuron marker; NF200, an A-fiber neuron marker. White Bar = 100 μm. White arrow represents double staining of P2X3 with IB4, white arrowhead represents double staining of P2X3 with NF200. [Reprinted from Wang et al. (2012). Copyright @ 2012 American Heart Association. Used with permission.]

**Figure 10:**
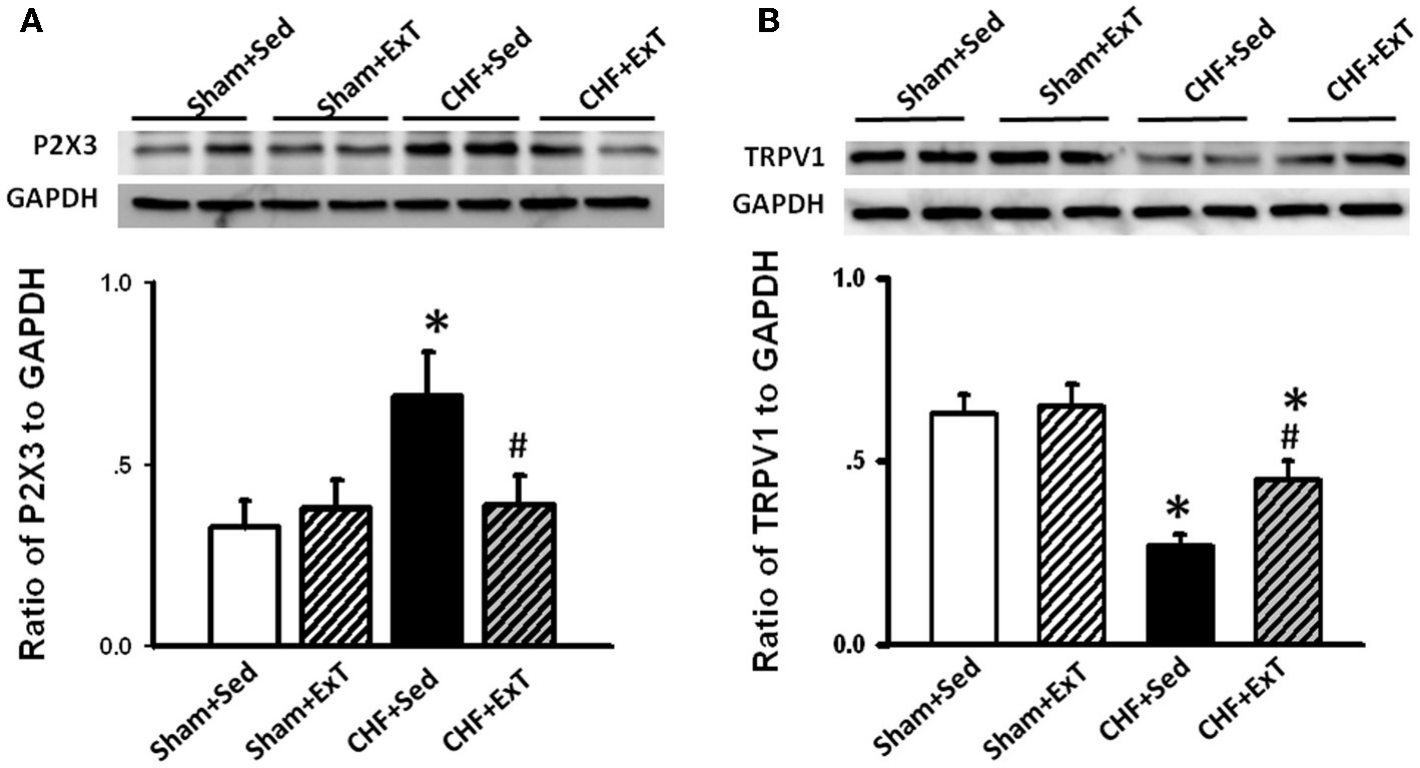
**Western blot data showing the protein expression of P2X3 (A) and TRPV1 (B) receptors in L4/L5 dorsal root ganglion (DRG) in Sham+Sed, Sham+ExT, CHF+Sed, and CHF+ExT rats.** Data are expressed as Mean ± SE. *n* = 6/each group. ^*^P < 0.05 vs. sham+Sed, #*P* < 0.05 vs. CHF+Sed. [Reprinted from Wang et al. (2012). Copyright @ 2012 American Heart Association. Used with permission.]

Purinergic (P) ligand-gated ion channels have been localized to both group III and IV muscle afferent neurons (Vulchanova et al., 1996, 1997, 1998). Skeletal muscle contraction triggers the release of purines such as adenosine and interstitial ATP, which act as ligands for P1 and P2X receptors, respectively (Hellsten et al., 1998; Li et al., 2003, 2005). Previous studies (Costa and Biaggioni, 1994; Middlekauff and Chiu, 2004) demonstrated that ATP is a potential metabolic stimulator of the EPR via the P2X receptor whereas adenosine and the P1 receptor is not involved in the modulation of the EPR. For example, an intra-arterial administration of α,β-methylene ATP (a P2X receptor agonist) into the hindlimb of decerebrated cats elevates BP and enhances afferent impulses from group IV fibers by 67% (Hanna and Kaufman, 2004). Furthermore, the arterial administration of the P2X receptor antagonist pyridoxal phosphate-6-azophenyl-2′, 4′-disulfonic acid (PPADS) attenuates the pressor response to static muscle contraction by 38% in cats and reduces the pressor response to post-contraction circulatory occlusion (Hanna and Kaufman, 2003). Furthermore, ATP may function as a metabolite that sensitizes mechanoreceptors. Kindig et al. (2006) reported that PPADS attenuated the responses of group III muscle afferents to static contraction as well as to tendon stretch in decerebrate cats, suggesting that P2 activation sensitizes group III afferents in the normal state. Recently, we (Wang et al., 2010a) demonstrated that (1) PPADS attenuated the response of group III muscle afferents to either static contraction or passive stretch in CHF rats to a greater extent than in sham rats; (2) protein expression of P2X3 receptors in the DRG was significantly increased in CHF rats compared with sham rats; (3) increased protein expression of P2X3 receptors in DRG was located on both IB4-positive (C fiber marker) and NF200-positive (A fiber marker) neurons. These findings suggest that ATP and P2X receptors are involved in the mechanism underlying the sensitization of group III afferents in CHF state. Furthermore, we (Wang et al., 2012) found that (1) the increased antagonistic effect of PPADS on the sensitivity of group III afferents observed in CHF rats was prevented by ExT (Figure 8) and (2) ExT prevented the upregulation of P2X3 receptors in both A- and C-fiber DRG neurons in CHF rats (Figures 9 and 10), indicating that ExT prevented the sensitization of group III afferents, at least in part, by the normalization of the upregulated P2X receptors in the CHF state.

### The TRPV1 receptor is involved in the mechanism by which ExT prevents the desensitization of group IV afferents in CHF

**Figure 11:**
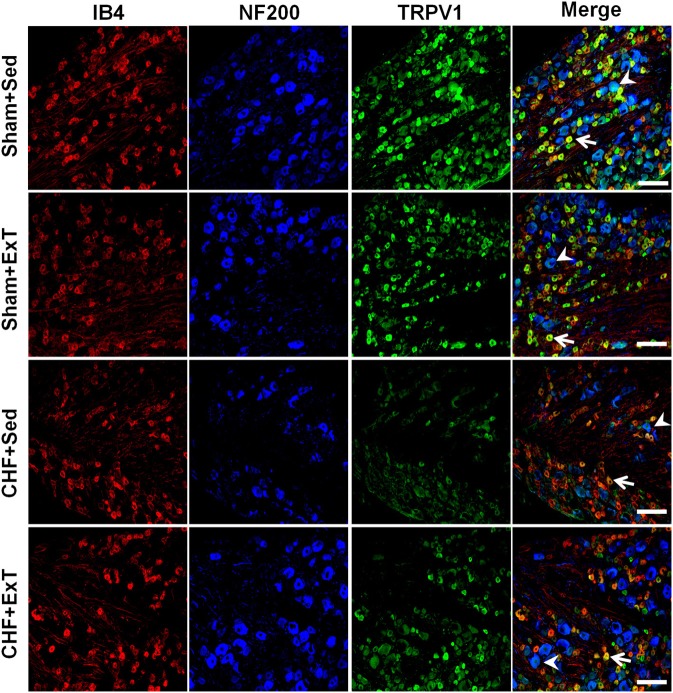
**Immunohistochemical data showing the protein expression of TRPV1 receptors in L4/L5 dorsal root ganglion (DRG) in sham and CHF rats.** IB4, a C-fiber neuron marker; NF200, an A-fiber neuron marker. White Bar = 100 μm. White arrow represents double staining of TRPV1 with IB4, white arrowhead represents double staining of TRPV1 with NF200. [Reprinted from Wang et al. (2012). Copyright @ 2012 American Heart Association. Used with permission.]

Transient receptor potential vanilloid 1 (TRPV1) receptors are predominantly localized to group IV fibers (Michael and Priestley, 1999; Wang et al., 2010a, 2012). Intra-arterial injection of capsaicin, a TRPV1 receptor agonist, markedly increases BP, HR, and SNA by stimulating group IV afferents (Crayton et al., 1981; Kaufman et al., 1982, 1983; Wang et al., 2010a,b). TRPV1 receptors are sensitive to changes in muscle temperature, increases in extracellular hydrogen ion concentration (pH <5.7), and inflammatory products such as bradykinin and prostaglandins (Tominaga et al., 1998; Guenther et al., 1999; Jordt et al., 2000; Welch et al., 2000). These potential activators of the TRPV1 receptor are present during exercise. An earlier study by Kindig et al. (2005) demonstrated that TRPV1 blockade failed to prevent the pressor response to static contraction in decerebrated cats, indicating that TRPV1 plays little role in evoking the EPR. On the contrary, Smith et al. (2010) recently reported that TRPV1 blockade attenuated the pressor response to static contraction in decerebrate rats, indicating that TRPV1 plays an important role in evoking the EPR. The discrepancy among studies is not readily apparent. However, the majority of studies do raise the possibility that the activation of TRPV1 receptors by skeletal muscle metabolites (e.g., protons) may contribute to the excitation of the skeletal muscle metaboreflex during exercise. In CHF animals, several reports by Smith and colleagues (Smith et al., 2005b, 2006a, 2010) demonstrated that (1) TRPV1 activation by capsaicin caused a blunted cardiovascular response in CHF rats compared to sham rats, which was confirmed by our recent study (Wang et al., 2010b); (2) chronic deletion of TRPV1 receptors in normal rats recapitulates the exaggerated EPR observed in CHF rats, indicating that the loss of TPRV1 receptors may be an important contributor to the development of the exaggerated EPR in CHF; and (3) the mRNA level of TRPV1 in the DRG and in skeletal muscle was decreased in CHF rats compared to sham rats. Recently, we (Wang et al., 2010a) further demonstrated that (1) the response of group IV afferents to exogenous TRPV1 activation by capsaicin was blunted in CHF rats and (2) protein expression of TRPV1 receptors in the DRG was significantly decreased in C-fiber DRG neurons of CHF rats. These findings suggest that the TRPV1 receptor plays an important role in causing the blunted group IV sensitivity in the CHF state. More recently, we (Wang et al., 2012) demonstrated that ExT partially prevents the blunted sensitivity of group IV afferents in response to either static contraction or to administration of capsaicin in CHF rats (Figure 7). This was associated with an improvement in the decrease in protein expression of TRPV1 receptors in C-fiber DRG neurons of CHF+ExT rats (Figures 10 and 11). These findings indicate that ExT improves the blunted sensitivity of group IV afferents, in part, by preventing the downregulation of TRPV1 receptors in muscle afferent neurons in the CHF state. It should be noted that similar to TRPV1 receptors, acid-sensing ion channels (ASICs), which open when exposed to an extracellular pH of 7.0 or less, are also localized to group IV fibers and contribute to the metaboreceptor component of the EPR (Chen et al., 1998; Zhang and Canessa, 2001; McCord et al., 2009). However, the role of ASIC channels in mediating the blunted metaboreflex as well as the desensitization of group IV afferents in CHF is largely unknown. Whether ASIC channels are involved in the ExT-induced improvement of the blunted metaboreflex as well as the densitization of group IV afferents in CHF remains unclear.

### Other potential mechanisms

Other peripheral mechanisms may play a role in mediating thebeneficial effects of ExT on reversing abnormal muscle afferent activity in CHF. For example, due to underperfusion of skeletal muscle in CHF, there is release of reactive oxygen species and inflammation. Augmented ROS production is strongly associated with endothelial dysfunction and may contribute to the exaggerated sympatho-excitatory response to exercise in CHF (Thomas et al., 2001; Thomas and Segal, 2004). In normal rats, we demonstrated that hindlimb infusion of a superoxide dismutase inhibitor increased ROS production within the skeletal muscle and augmented the pressor response to static muscle contraction (Wang et al., 2009). This sympatho-excitatory response was significantly attenuated by intra-arterial infusion of either a superoxide dismutase mimetic Tempol or an NADPH-oxidase inhibitor apocynin (Wang et al., 2009), indicating that ROS plays an excitatory role in modulation of the EPR. We recently demonstrated (Wang et al., 2011) that the both Tempol and a membrane permeable superoxide dismutase, polyethylene glycol-superoxide dismutase (PEG-SOD) attenuated sodium channel activity in muscle afferent neurons in rats. Because we provided evidence that sodium channels in muscle afferent neurons are critical for the genesis of the EPR (Wang et al., 2011), the inhibitory effect of ROS scavengers (Tempol and PEG-SOD) on sodium channel activity in muscle afferent neurons indicates that ROS modulates the EPR by affecting sodium channel activity in muscle afferents. In contrast, the studies from Koba et al. (2009) and McCord et al. (2011) found that injection of Tempol into hindlimb and trapping the circulation to maximize the local effects of the drug were unable to verify that Tempol attenuated the pressor response to static contraction in normal rats, indicating that local ROS in skeletal muscle did not modulate the EPR in normal state. However, it should be noted that these investigators did not measure the EPR function immediately after stopping the trap protocol but rather during a 30-min of reperfusion after trapping. This may exhaust or minimize the pharmacologic effect of Tempol by either dynamic metabolism or ischemic-reperfusion. Therefore, whether local ROS in skeletal muscle is involved in the modulation of the EPR in the normal state is still controversial. Direct evidence from muscle afferent recording is needed to address this discrepancy. However, in rats with CHF induced by myocardial infarction, the entrapment of Tempol within the hindlimb circulation did produce a marked reduction in BP, HR, and renal SNA in response to the activation of the EPR (Koba et al., 2009). Collectively, these data suggest that increases in ROS generation (oxidative stress) in the hindlimb skeletal muscle contributes to the exaggerated cardiovascular response to stimulation of the EPR in CHF. With regard to the antioxidant and anti-inflammatory effects of ExT (Linke et al., 2005; Batista et al., 2010) in CHF, it is reasonable to speculate that the decreased muscle ROS level by ExT may bring the exaggerated EPR close to normal. Clearly, further research is needed in this area.

## Future directions

The exaggerated sympatho-excitation during exercise potentially increases cardiovascular risk and contributes to exercise intolerance during physical activity in CHF patients (Grassi and Mancia, 1999; Piepoli et al., 1999; Smith et al., 2006a). A therapeutic strategy for preventing or slowing the progression of the exaggerated EPR may significantly improve symptoms of exercise intolerance and reduce cardiovascular risk in CHF patients. This review has summarized the evidence from human and animal experiments suggesting that that long-term ExT has a beneficial effect on the exaggerated EPR in the CHF state. Evidence from our animal studies indicates that ExT in the early stage of CHF (2 weeks after coronary ligation) has a “protective” effect (rather than a “curative” effect) on the exaggerated EPR-evoked sympatho-excitation during exercise in CHF since the exaggerated EPR has not developed (Smith et al., 2003) at that time point. In addition, this training strategy also has a protective effect on the elevated resting sympathetic tone in CHF. An important clinical relevance of these findings is that patients recovering from myocardial infarction can take advantage of this early ExT strategy to slow or improve the symptoms associate with CHF. However, whether the benefits of this strategy can be applied for all degrees of CHF patients is unclear. Clearly, further studies are necessary for to determine which CHF patients will derive maximal benefit from ExT. It will also be important to determine what dose (i.e., duration and intensity) of ExT can be tolerated safely in CHF patients and still be effective. Furthermore, whether ExT can improve functional capacity of patients with more established CHF and an exaggerated EPR remains unclear. Piepoli et al. (1996) reported that 6-weeks of forearm training partially reversed the exaggerated exercise-evoked sympatho-excitation, vasoconstrictor response and ventilatory drive in patients with CHF. Whether central command is also involved in these ExT-mediated benefits remains largely unknown. Further animal studies are necessary to isolate the contribution of central command to the ExT-mediated benefits during exercise.

### Conflict of interest statement

The authors declare that the research was conducted in the absence of any commercial or financial relationships that could be construed as a potential conflict of interest.
